# Lunasin attenuates obesity-related inflammation in RAW264.7 cells and 3T3-L1 adipocytes by inhibiting inflammatory cytokine production

**DOI:** 10.1371/journal.pone.0171969

**Published:** 2017-02-09

**Authors:** Chia-Chien Hsieh, Mei-Jia Chou, Chih-Hsuan Wang

**Affiliations:** Department of Human Development and Family Studies, National Taiwan Normal University, Taipei, Taiwan; Universidad Pablo de Olavide, SPAIN

## Abstract

Obesity has become a major threat to public health and is accompanied by chronic low-grade inflammation, which leads to various pathological developments. Lunasin, a natural seed peptide, exhibits several biological activities, such as anti-carcinogenesis, anti-inflammatory, and antioxidant activities. However, the mechanism of action of lunasin in obesity-related inflammation has not been investigated. The aim of this study was to explore whether lunasin could reduce the inflammation induced by obesity-related mediators in RAW264.7 cells and 3T3-L1 adipocytes and whether it could attenuate the crosstalk between the two cell lines. RAW264.7 cells were cultured in leptin-containing medium, adipocyte-conditioned medium (Ad-CM), or co-cultured with 3T3-L1 cells to mimic the physiology of obesity. The data showed that the secretion of pro-inflammatory cytokine interleukin-1β (IL-1β) was inhibited by lunasin after leptin activation of RAW264.7 cells. In addition, lunasin decreased monocyte chemoattractant protein-1 (MCP-1) and IL-1β secretions in the Ad-CM model. Cytokine MCP-1, IL-6, tumor necrosis factor (TNF)-α, and IL-1β secretions were significantly decreased by leptin or Ad-CM plus lipopolysaccharide stimulation. Subsequently, the co-culture of the two cells refined the direct relation between them, resulting in apparently increased MCP-1, and decreased IL-6 levels after lunasin treatment. In 3T3-L1 adipocytes, lunasin also exhibited anti-inflammatory property by inhibiting MCP-1, plasminogen activator inhibitor-1, and leptin productions stimulated by (TNF)-α, lipopolysaccharide, or RAW264.7 cell-conditioned medium. This result revealed that lunasin acts as a potential anti-inflammatory agent not only in macrophages but also in adipocytes, disrupting the crosstalk between these two cells. Therefore, this study suggests the intake of lunasin from diet or as a supplement, for auxiliary prevention or therapy in obesity-related inflammatory applications.

## Introduction

Statistical estimation by the World Health Organization in 2014 indicated that 39% of the adults worldwide were overweight and 13% were clinically obese, approximating to a total of 2.1 billion people worldwide [1]. Adipose tissue plays a major endocrine role of secreting various adipokines that affect the physiology [2]. However, excessive accumulation of energy transfer to adipocytes due to hyperplasia and hypertrophy is termed as obesity. Obesity is characterized by low-grade inflammation of the microenvironment with infiltration by various immune cells, such as leukocytes, granulocytes, monocytes/macrophages, lymphocytes, and dendritic cells, which overproduce a series of pro-inflammatory and pro-atherogenic mediators [3, 4]. Therefore, obesity initiation and development are linked to several obesity-associated diseases, such as cardiovascular complications, metabolic disorders [4], and several types of cancers [5, 6].

In obesity, the main players in the systemic chronic inflammation are the increased numbers of pro-inflammatory macrophages and production of deregulated hormones and cytokines, such as monocyte chemoattractant protein-1 (MCP-1), interleukin-6 (IL-6), IL-1β, and tumor necrosis factor-α (TNF-α), by the adipose tissue [2]. Particularly, this inflammation process involves regulation of various cells stimulating the production of recruited chemokines and active cytokines, to modulate the signaling pathways of energy and lipid metabolism, insulin resistance, cell proliferation in the microenvironment, and epigenetic genes expression [4].

Inflammation, as a pathophysiological condition, is involved in the development of many chronic diseases. A study has reported the potential benefits of supplementary diets and micronutrients that modulate the local and systemic chronic inflammation [7]. Therefore, food components are important mediators that participate in pro-inflammatory or anti-inflammatory reactions. Recently, several studies have demonstrated that food can be scored according to their inflammatory capacity, termed as dietary inflammatory index, which shows a close correlation of diet with inflammation and cardio-metabolic diseases [8].

Lunasin is a 43 amino acid-long natural peptide that was first identified in soybean [9], several grains, and herbal plants [10]. This peptide has been shown to exhibit biological activities against diseases, such as cancer, cardiovascular diseases, and immune disorders, in both *in vitro* and *in vivo* studies [10, 11]. In 2009, its anti-inflammatory property was first proposed. Moreover, it also shows antioxidant activity. Both these properties may contribute to its chemopreventive actions [12]. The anti-inflammatory property of lunasin has been demonstrated in RAW264.7 cells stimulated by lipopolysaccharide (LPS), resulting in the inhibition of pro-inflammatory cytokine production [12], possibly by blocking of the nuclear factor-κB (NF-κB) signaling pathway in RAW264.7 cells [13, 14] and by the down-regulation of Akt-mediated NF-κB activation in active THP-1 macrophages [15]. Lunasin leads to reduction in the inflammatory reaction induced by macrophages, through endocytic mechanisms involving clathrin-coated vesicles and macropinosomes [16].

Obesity provides an inflammatory microenvironment, which is favorable to metabolic complications and even tumorigenesis. Based on this evidence, dietary compounds have a major role in inflammation-related outcomes [7]. It is particularly intriguing to understand how lunasin operates in relation to the adipose microenvironment. However, only a few studies have explored natural compounds used to disrupt the crosstalk between macrophages and adipocytes, applying them to obesity-related inflammatory disorders. In the present study, we investigated the anti-inflammatory property of lunasin on RAW264.7 cells and 3T3-L1 adipocytes and set up models to explore their crosstalk. As the anti-inflammatory property of lunasin linked to adipose tissue inflammation holds great promise as a candidate for future therapeutic intervention, a better understanding of its underlying actions is required.

## Materials and methods

### Cell culture and reagents

Mouse RAW264.7 macrophages and 3T3-L1 fibroblasts were kindly provided by Dr. Tsai and Dr. Lin of National Taiwan Normal University and National Taiwan University (Taipei, Taiwan), respectively. RAW264.7 and 3T3-L1 cells were cultured in Dulbecco’s modified Eagle’s medium (DMEM; Caisson, Smithfield, UT, USA), containing fetal bovine serum (FBS; Genedirex, Las Vegas, NV, USA), 10% heat-inactivated calf serum (BS, Gibco, Grand Island, NY, USA), and 1% penicillin/streptomycin/amphotericin B (Caisson), and were incubated at 37°C in a humidified incubator with 5% CO_2_ atmosphere. These two cells from murine were chosen referred to many co-culture or conditional medium models in the present study. Cells from the same species are reasonable and easy to handle in the experimental operation. Lunasin, a 43 amino acid-long peptide with the sequence SKWQHQQDSCRKQLQGVNLTPCEKHIMEKIQGRGDDDDDDDDD, was chemically synthesized by KaiJie Bio-pharmaceutical Company (Chengdu, China). The purity of this peptide was greater than 95%.

### Cell viability assay

RAW264.7 cells were seeded at a density of 1 × 10^4^ cells/well in 96-well plates (Becton Dickinson, Franklin Lakes, NJ, USA) and were treated with various concentrations of lunasin, leptin (recombinant mouse leptin protein, R&D Systems, Minneapolis, MN, USA), and LPS (Sigma, St. Louis, MO, USA). After 24 h of the treatments, cells were incubated with 0.5 mg/mL 3-(4,5-dimethylthiazol-2-yl)-2,5-diphenyltetrazolium bromide (MTT; Sigma) solution for 3 h at 37°C, after which the supernatant was aspirated and dimethyl sulfoxide was added to solubilize the formazan crystals. The spectrophotometric absorbance at 540 nm was determined by a microplate reader (BioTek, Winooski, VT, USA). The cell viability was calculated as a percentage of control, which was considered as 100%, according to the formula: (A_sample_ − A_blank_)/(A_control_ − A_blank_) × 100.

### Differentiation of 3T3-L1 preadipocytes into adipocytes

The 3T3-L1 fibroblasts were differentiated to adipocytes as described previously [17]. Briefly, 3T3-L1 fibroblasts were seeded at 3×10^4^ cells/well in 24-well plates (Becton Dickinson) containing 10% FBS/DMEM. After the cells reached full confluence, differentiation was induced by using medium I: 25 mM glucose, 0.5 mM 3-isobutyl-1-methylxanthine (Sigma), 0.2 μM dexamethasone (Sigma), and 10 μg/mL insulin (Sigma) in 10% FBS medium, for the first 4 days and then by medium II: 25 mM glucose, 10 μg/mL insulin, and 10% FBS medium for 13 days. The medium was replaced by fresh medium II every 3 days to promote the maturation of adipocytes. At the end of the differentiation, the cells exhibited the adipocyte phenotype and they were then designed for further analyses.

### Culture of RAW264.7 cells in obesity-related inflammatory models

To test the effects of LPS, leptin, and adipocyte-conditioned medium (Ad-CM) on RAW264.7 cell growth and adipokine secretion, serial doses of the treatments were added to the cells. Cell numbers were counted using the MTT assay, and MCP-1 level was analyzed by ELISA. Then, the obesity-related inflammatory models were set up for further *in vitro* experiments.

#### Leptin-induced adipocyte inflammation

Cells were treated with serial doses of leptin or leptin plus 100 ng/mL LPS for 24 h. MCP-1 production was analyzed in the supernatant to find the optimal stimulated condition. Then, cells were treated with 1, 10, and 50 μM of lunasin and were activated by 200 ng/mL leptin, 100 ng/mL LPS, or a combination of both, for 24 h. Culture supernatants were collected for cytokine analysis by ELISA.

#### Culture of RAW264.7 cells in Ad-CM

Ad-CM was generated according to the previous protocol [17], the 3T3-L1 adipocytes differentiated on day 12, the medium was replaced by 10% FBS/DMEM for 24 h, and then the supernatant named Ad-CM was collected for subsequent *in vitro* study. RAW264.7 cells were seeded at 2×10^4^ cells/well in a 48-well plate, treated with various doses 1, 10, and 50 μM of lunasin, with or without LPS activation, and were cultured in 25% Ad-CM and 75% medium with 10% FBS for 24 h to mimic the environment of adipocytes around the macrophages. Culture supernatants were collected for adipokine analysis by ELISA.

#### Co-culture of 3T3-L1 adipocytes with RAW264.7 cells

To mimic the physiological environment where obesity triggers macrophage infiltration, the macrophages and adipocytes were co-cultured in a transwell culture system. The 3T3-L1 cells were seeded in a 24-well plate in the lower compartment of a transwell culture system (0.4 μm pore size; Costar, Kennebunk, MA USA) and differentiated into adipocytes until day 13. The RAW264.7 cells were seeded at 4×10^4^ cells/well in the upper chamber of the transwell. Then, both cells were put together in the co-culture system maintained in 10% FBS/DMEM and treated with 10 and 50 μM lunasin for 24 h. Culture supernatants were collected and stored at −20°C for cytokine assay.

### 3T3-L1 adipocyte inflammatory models

#### LPS- and TNF-α-induced adipocyte inflammation

3T3-L1 cells were seeded at 3×10^4^ cells/well in a 24-well plate for differentiation into adipocytes. On day 13, 3T3-L1 adipocytes were treated with various doses 5, and 25 μM of lunasin and were stimulated by 1000 ng/mL LPS or 10 ng/mL TNF-α (PeproTech, Rocky Hill, NJ, USA) in 1% FBS/DMEM simultaneously at the same time for 24 h. Culture supernatants were collected for inflammatory cytokine and adipokine analysis by ELISA.

#### RAW264.7 Cell-conditioned Medium (RAW-CM)-induced adipocyte inflammation

RAW264.7 cells were plated at a density of 5×10^5^ cells/well in 24-well plates containing 10% FBS/DMEM, overnight. The cells were stimulated with 100 ng/mL LPS in 1% FBS/DMEM for 24 h, and the supernatants were collected and centrifuged to remove cell debris, for subsequent *in vitro* studies. 3T3-L1 cells were treated with various doses 5, and 25 μM of lunasin and were cultured in 50% RAW-CM containing 1% FBS/DMEM for 24 h. Culture supernatant was collected for cytokine and adipokine assay.

### Determination of cytokine and adipokine production by ELISA

RAW264.7 and 3T3-L1 cells were seeded at 2×10^4^ cells/well in a 48-well plate and 3×10^4^ cells/well in a 24-well plate, respectively, overnight. Cells were treated with lunasin using various models for 24 h. Culture supernatants were collected and cytokines, such as IL-6, MCP-1, leptin, adiponectin, and plasminogen activator inhibitor-1 (PAI-1), were analyzed by ELISA, according to the manufacturer’s protocols (R&D Systems). In brief, capture antibodies, cultured supernatants, detection antibodies, streptavidin conjugated horseradish-peroxidase were processed on the plate in order, and the color subtract tetramethylbenzidine (R&D Systems) was used. The absorbance was measured and the concentration was calculated according to the standard.

### Statistical analysis

The results were analyzed form at least three independent experiments and presented as mean ± standard error of mean (SEM). Differences between the groups were analyzed by one-way ANOVA, followed by least significant difference (LSD) test using IBM Statistical Product and Service Solutions (SPSS) version 19. *P*-value less than 0.05 was considered statistically significant.

## Results

### Experimental design and basal conditions

**Fig 1:**
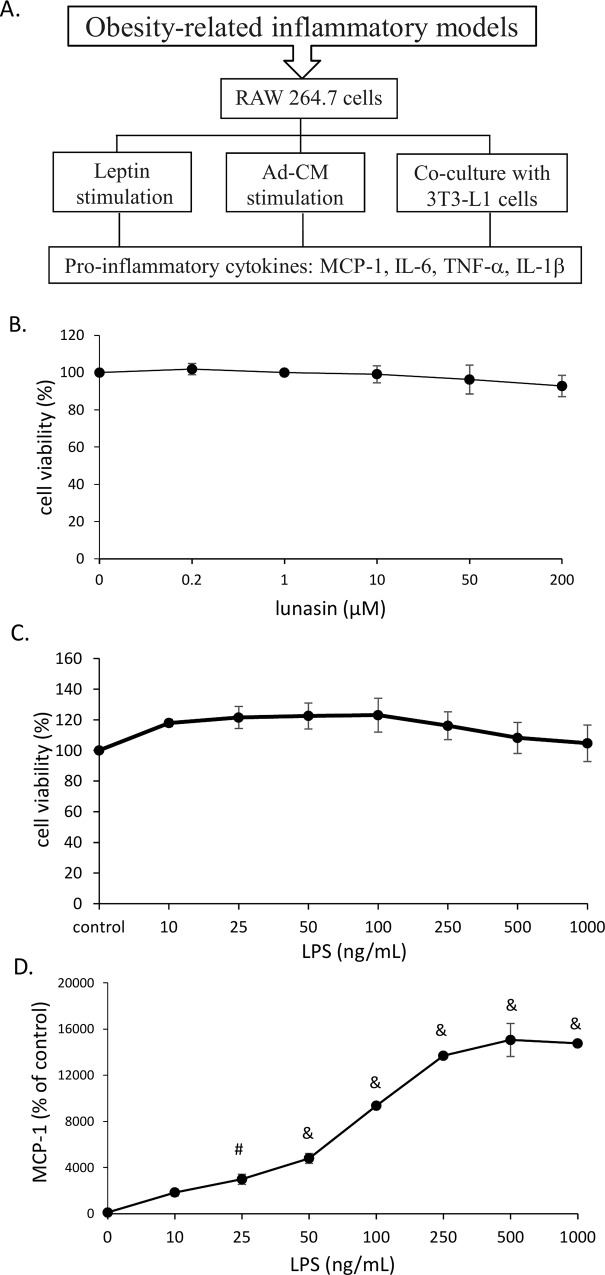
Experimental design and basal conditions. **A**. The concept of experimental designs included leptin supplement, Ad-CM replacement, and co-culture system. **B**. RAW264.7 cells were treated with a serial dose of lunasin for 24 h and cell proliferation was tested by MTT assay. **C.** RAW264.7 cells were treated with a serial dose of LPS for 24 h and cell proliferation was tested by MTT assay. **D**. RAW264.7 cells were treated with various concentrations of LPS for 24 h, and MCP-1 production in culture supernatant was determined by ELISA. Data are presented as mean ± SEM, from three independent experiments. Statistical analysis was based on one-way ANOVA and LSD post hoc test; # *p* < 0.001 or & *p* < 0.005 *vs*. control group. Ad-CM, adipocyte-conditioned medium; MCP-1, monocyte chemoattractant protein-1; TNF-α, tumor necrosis factor-α; IL, interleukin.

To mimic the physiological environment of obesity associated with inflammation due to macrophage infiltration, three *in vitro* models: 1) leptin-treated macrophages, 2) Ad-CM-treated macrophages, and 3) co-culture of adipocytes and macrophages, were built to study the effect of lunasin on the obesity-related inflammatory reaction in RAW264.7 macrophages (Fig 1A).

Treatment with various concentrations of lunasin (0.2 to 200 μM) did not affect the growth of RAW264.7 cells (Fig 1B), suggesting that lunasin was a safe and non-toxic natural component. Subsequently, the effect of LPS treatment (10 to 1000 ng/mL) on RAW264.7 cells was tested using MTT assay, to confirm that the following effects were not due to changes in the cell numbers (Fig 1C). MCP-1 secretion increased with rise in LPS dosage, and this production saturated on activation with 500 ng/mL LPS (Fig 1D). A middle dose of 100 ng/mL LPS, which causes mild activation of macrophages, was used as a condition for future *in vitro* experiments.

### Lunasin inhibited inflammatory cytokine production in leptin-conditioned RAW264.7 cells

**Fig 2:**
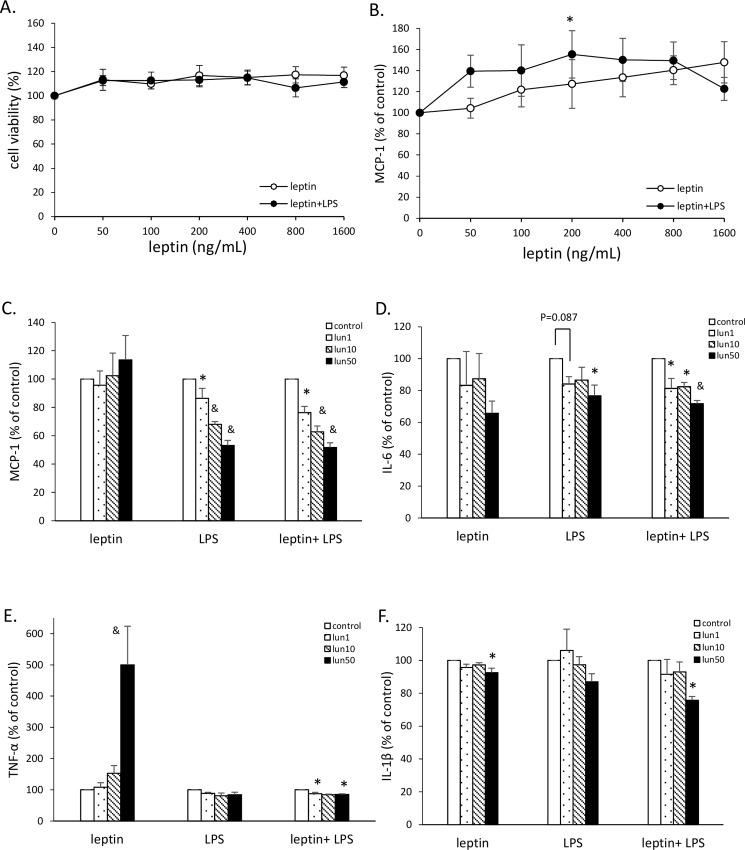
Lunasin inhibited inflammatory cytokine production in leptin-conditioned RAW264.7 cells. **A**. Cell proliferation of RAW264.7 cells was determined by MTT assay. Cells were treated with serial doses of leptin or leptin plus 100 ng/mL LPS for 24 h. **B**. MCP-1 secretion by RAW264.7 cells was analyzed by ELISA. Cells were treated with various concentrations of leptin in the presence or absence of 100 ng/mL LPS for 24 h. **C**. MCP-1 production after 24 h incubation was analyzed in the culture supernatant by ELISA. Cells were treated with various concentrations of lunasin and were activated by 200 ng/mL leptin, 100 ng/mL LPS, or a combination of both, for 24 h. **D**. IL-6 production was analyzed by ELISA. **E**. TNF-α production was analyzed by ELISA. **F**. IL-1β production was analyzed by ELISA. Data are presented as mean ± SEM, from three independent experiments. Statistical analysis was based on one-way ANOVA and LSD post hoc test; * *p* < 0.05, # *p* < 0.001, or & *p* < 0.005 *vs*. control group. LPS, lipopolysaccharide; MCP-1, monocyte chemoattractant protein-1; lun, lunasin; IL, interleukin; TNF-α, tumor necrosis factor-α.

Several studies have demonstrated the significantly high level of leptin in obese subjects [18]. Therefore, we added recombinant mouse leptin protein to the medium to indirectly mimic the physiological conditions in obesity. The cell growth and MCP-1 secretion by RAW264.7 cells were analyzed after treatment with serial doses of leptin, with or without LPS stimulation. The leptin treatments at doses 50 to 1600 ng/mL did not affect cell viability compared to the untreated control group (Fig 2A). MCP-1 secretion gently increased with an increase in leptin concentration, and the secretion was significantly high at 200 ng/mL leptin with LPS stimulation (Fig 2B). Therefore, the leptin concentration of 200 ng/mL was used as a condition for future experiments.

To investigate whether lunasin affected pro-inflammatory cytokine secretion from macrophages in obesity-related environments, RAW264.7 cells were treated with 1, 10, and 50 μM lunasin and were activated with leptin, LPS, or a combination of both, to induce an obesity-related inflammatory reaction. Leptin activation provided a mild stimulation; therefore, LPS supplement was used to enhance the inflammatory activation of RAW264.7 macrophages. In the active models, production of pro-inflammatory cytokines MCP-1, IL-6, TNF-α, and IL-1β apparently increased compared to that in non-activated cells (Fig 2C–2F). In the LPS stimulation model of RAW264.7 cells, treatments with 1, 10, and 50 μM lunasin showed a dose-dependent decrease of 14, 32, and 47%, respectively, in MCP-1 secretion (p = 0.039, p < 0.005, p < 0.005, respectively; Fig 2C) and a significant decrease of 23% in IL-6 secretion at 50 μM lunasin concentration (p = 0.017; Fig 2D). In the leptin plus LPS model, lunasin treatments (1, 10, and 50 μM) showed a dose-dependent decrease of 24, 37, and 48% in MCP-1 and 19, 18, and 28% in IL-6 secretions, respectively (p < 0.05; Fig 2C and 2D), and a significant decrease in TNF-α and IL-1β secretions at a concentration of 50 μM lunasin (p = 0.018, p = 0.024, respectively; Fig 2E and 2F). In the model with leptin alone as a stimulator, this suppressive property of lunasin was reflected only by a decrease in IL-1β secretion, which may be due to the very mild activation by leptin.

### Lunasin inhibited inflammatory cytokine production in Ad-CM-conditioned RAW264.7 cells

**Fig 3:**
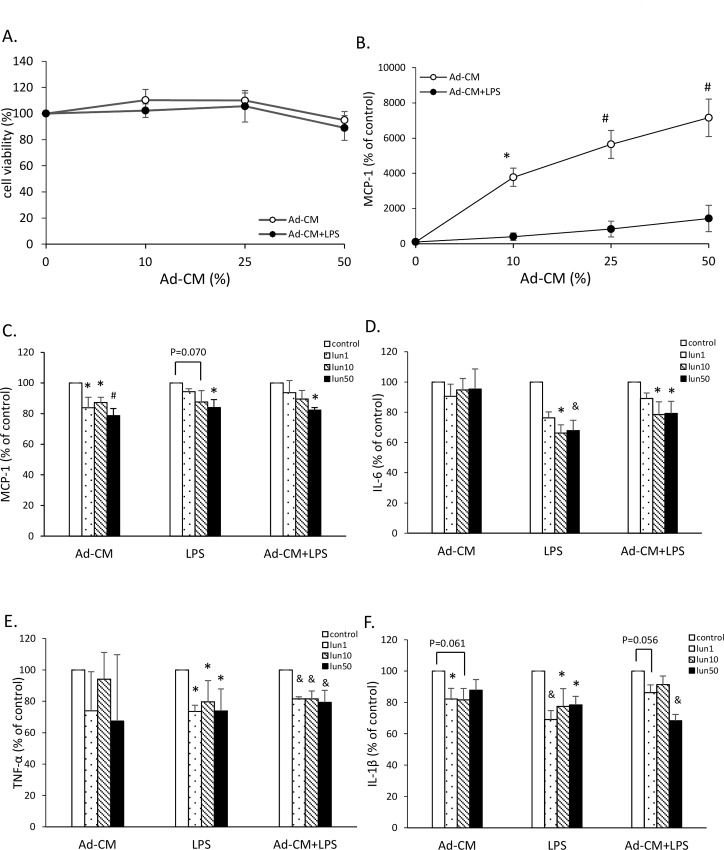
Lunasin inhibited inflammatory cytokine production in Ad-CM-conditioned RAW264.7 cells. **A**. Cell proliferation of RAW264.7 cells was determined by MTT assay. Cells were cultured in the presence of increasing concentration of Ad-CM for 24 h. **B**. MCP-1 production by RAW264.7 cells was analyzed by ELISA. Cells were treated with multiple doses of Ad-CM in the presence or absence of 100 ng/mL LPS for 24 h. **C**. MCP-1 production after 24 h culture was analyzed in the supernatant by ELISA. Cells were treated with multiple doses of lunasin and were activated by 25% Ad-CM, 100 ng/mL LPS, or a combination of both for 24 h. **D**. IL-6 production was analyzed by ELISA. **E**. TNF-α production was analyzed by ELISA. **F**. IL-1β production was analyzed by ELISA. Data are presented as mean ± SEM from three independent experiments. Statistical analysis was based on one-way ANOVA and LSD post hoc test; * *p* < 0.05, # *p* < 0.001, or & *p* < 0.005 *vs*. control group. Ad-CM, adipocyte-conditioned medium; LPS, lipopolysaccharide; MCP-1, monocyte chemoattractant protein-1; lun, lunasin; IL, interleukin; TNF-α, tumor necrosis factor-α.

To evaluate the anti-inflammatory actions of lunasin in the obesity-related environment, the effects of inflammatory mediators were analyzed in the culture supernatant. Ad-CM was generated to mimic the physiology of obesity, according to a previous study [17]. In this model, RAW264.7 cells were cultured in replaced Ad-CM medium to stimulate obesity-related inflammation in the macrophages (Fig 3). The control group was defined as 0% Ad-CM, i.e., cell culture without conditioned medium. After being cultured in 10, 25, and 50% Ad-CM in the presence or absence of LPS stimulation for 24 h, the growth and MCP-1 secretion by RAW264.7 cells were analyzed. The cell viability was not affected by Ad-CM treatment (Fig 3A), but the MCP-1 secretion gently increased with increase in the dose of Ad-CM, compared to that in the control medium, especially on LPS stimulation (p < 0.05; Fig 3B). The middle dose of Ad-CM at 25%, which caused mild activation, was used as a condition for future *in vitro* experiments.

The cells were treated with 1, 10, and 50 μM lunasin and were activated by Ad-CM, LPS, or a combination of both to induce an obesity-related inflammatory reaction. Cytokine MCP-1, IL-6, TNF-α, and IL-1β productions apparently increased in active models, compared to those with use of medium alone (Fig 3). In the model with Ad-CM, the suppressive property of lunasin was reflected by decrease in MCP-1 and IL-1β secretions. MCP-1 secretion, by the cells treated with 1, 10, and 50 μM lunasin, significantly decreased by 16, 13, and 21%, respectively (p < 0.05; Fig 3C), and IL-1β production decreased by 18, 18, and 12%, respectively (p = 0.015, p = 0.061, p = 0.090, respectively; Fig 3F) compared to that in the control group. In the LPS stimulation model of RAW264.7 cells, 50 μM lunasin decreased MCP-1 secretion by 16% (p = 0.024; Fig 3C). Treatment with 10 and 50 μM lunasin significantly decreased the secretion of IL-6 by 34 and 32%, TNF-α by 20 and 26%, and IL-1β by 22 and 22%, respectively (p < 0.05; Fig 3D–3F). In the Ad-CM plus LPS model, 50 μM lunasin treatment led to decrease in MCP-1 secretion (p = 0.017) and significant decrease in IL-6 and IL-1β productions (p < 0.05; Fig 3D and 3F). In addition, it significantly decreased TNF-α secretion at all concentrations (p < 0.005; Fig 3E).

### Lunasin disrupted the crosstalk between macrophages and adipocytes

**Fig 4:**
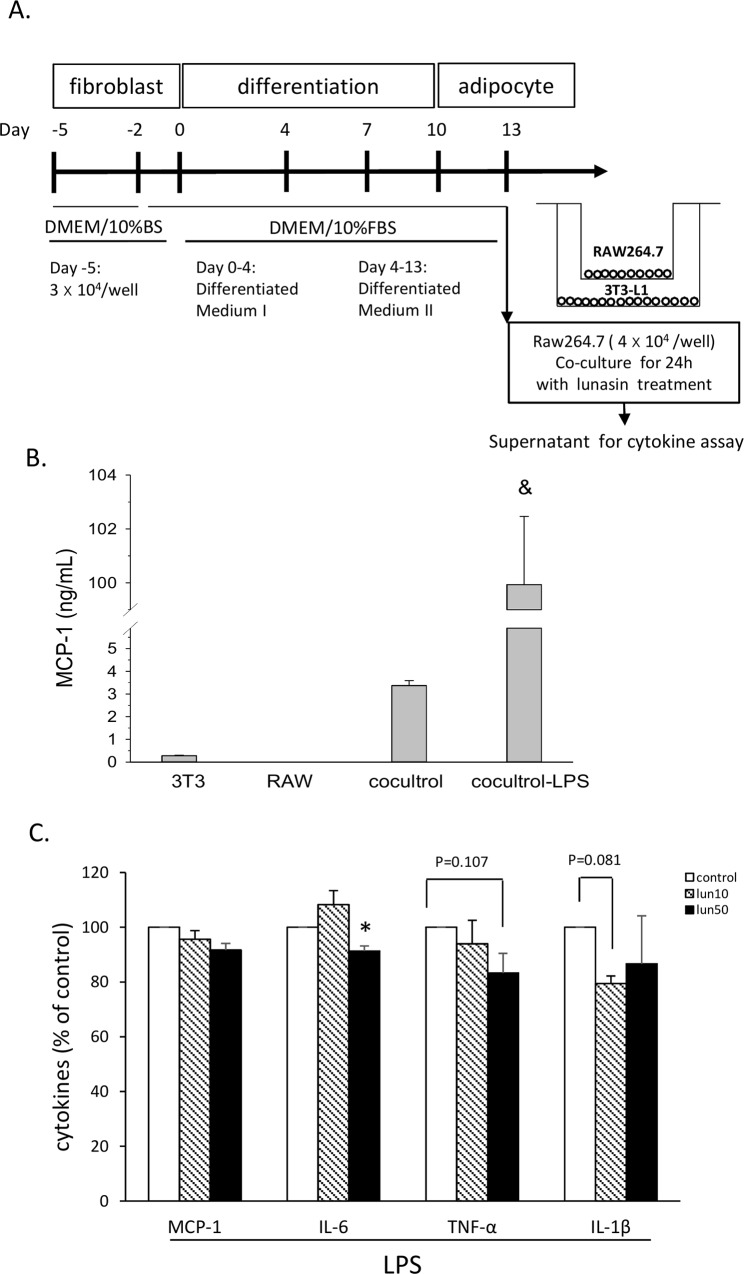
Lunasin abated inflammatory cytokine production in the RAW264.7 cell and mature 3T3-L1 adipocyte co-culture. **A.** RAW264.7 cells were co-cultured with mature adipocytes using a transwell system for 24 h. **B.** MCP-1 level in the supernatant obtained from 3T3-L1 cell culture, RAW264.7 cell culture, and co-culture of both cells in the presence or absence of 100 ng/mL LPS was measured by ELISA. **C**. MCP-1, IL-6, TNF-α, and IL1-β secretions in the supernatant obtained from the 24 h co-culture were measured by ELISA. Data are presented as mean ± SEM, from three independent experiments. Statistical analysis was based on one-way ANOVA and LSD post hoc test; * *p* < 0.05 *vs*. control group. DMEM, Dulbecco’s modified Eagle’s medium; BS, calf serum; FBS, fetal bovine serum; MCP-1, monocyte chemoattractant protein-1; LPS, lipopolysaccharide; lun, lunasin; IL, interleukin; TNF-α, tumor necrosis factor-α.

Potential mediators of interactions between RAW264.7 cells and 3T3-L1 adipocytes were evaluated by a co-culture transwell system for 24 h (Fig 4A). The level of MCP-1 in the medium with only 3T3-L1 or RAW264.7 cells was very low, but increased by four times in the supernatant of co-culture of both cells. MCP-1 level significantly was increased by approximately 26-fold upon LPS stimulation, compared to that in the co-culture without LPS stimulation (Fig 4B). The culture supernatant was analyzed at the end of the co-culture period, and the levels of cytokines MCP-1, IL-6, TNF-α, and IL1-β were measured by ELISA. Data showed that these cytokine secretions by the cells were stimulated by LPS (Fig 4C), as these cytokine levels were not significant among groups without stimulation (data not shown). When both cell types were present, the IL-6 production was reduced by 9% (p < 0.05) on treatment with 50 μM lunasin, whereas a decrease in TNF-α (17%) and IL-1β (21%) secretions was observed on 50 μM and 10 μM lunasin treatments (p = 0.107, p = 0.081, respectively; Fig 4C). The results of this model were not significant like those of leptin and Ad-CM models, suggesting that the co-culture system was more complicated and the culture conditions should be improved to get an appropriate response. This data also indicated that lunasin treatment blocked the production of mediators related to inflammatory reaction in the co-culture system.

### Lunasin inhibited pro-inflammatory adipokine production in 3T3-L1 adipocytes

**Fig 5:**
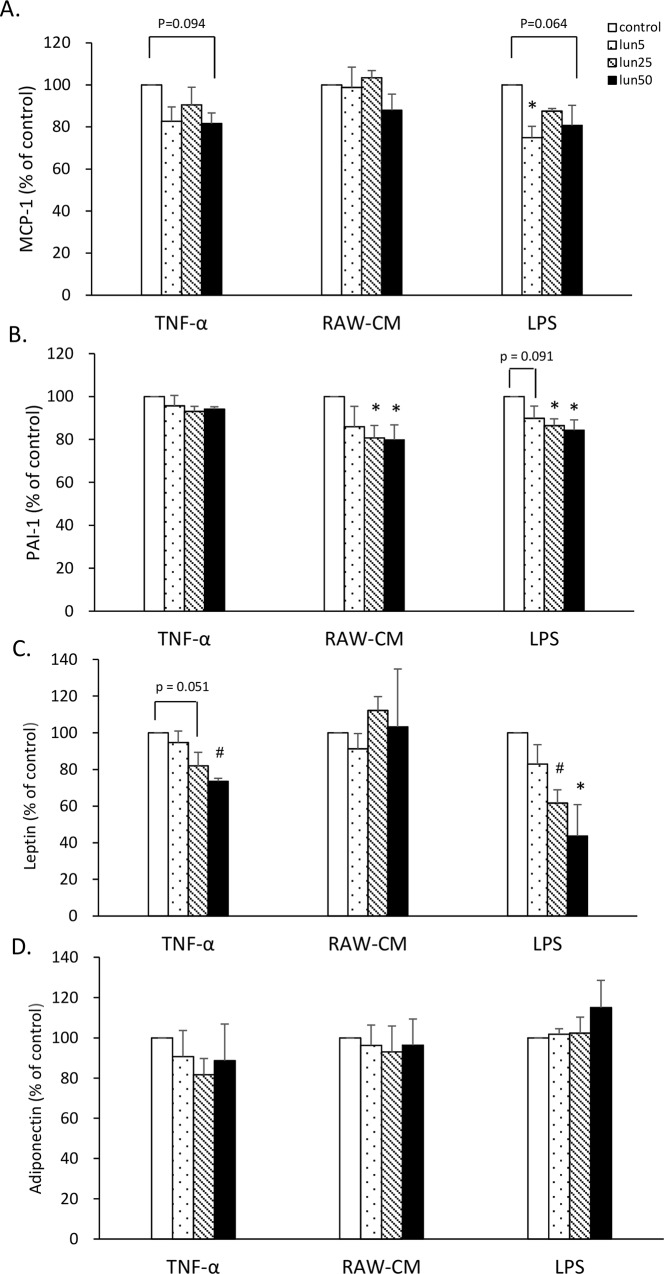
Lunasin inhibited pro-inflammatory adipokine production in 3T3-L1 adipocytes. 3T3-L1 adipocytes were treated with various doses of lunasin and were stimulated with 10 ng/mL TNF-α, 50% RAW-CM, or 1 μg/mL LPS to induce inflammatory conditions for 24 h. **A.** MCP-1 production was analyzed in the supernatant by ELISA. **B.** PAI-1 production was analyzed by ELISA. **C.** Leptin production was analyzed by ELISA. **D.** Adiponectin production was analyzed by ELISA. Data are presented as mean ± SEM from three independent experiments. Statistical analysis was based on one-way ANOVA and LSD post hoc test; * p < 0.*05*, *or # p < 0*.*001* vs. control group. LPS, lipopolysaccharide; MCP-1, monocyte chemoattractant protein-1; lun, lunasin; IL, interleukin; PAI-1, plasminogen activator inhibitor-1; RAW-CM, RAW264.7 cell-conditioned medium; TNF-α, tumor necrosis factor-α.

To evaluate the anti-inflammatory activity of lunasin on 3T3-L1 adipocytes, the effects on adipokines were analyzed. Lunasin did not affect the differentiation and lipid accumulation of 3T3-L1 adipocytes (data not shown). 3T3-L1 cells were treated with 5, 25, and 50 μM lunasin and were stimulated with 10 ng/mL TNF-α, 50% RAW-CM, or 1 μg/mL LPS to induce inflammatory conditions in the adipocytes (Fig 5). In the LPS stimulation model, the MCP-1 level decreased by 25% (p = 0.022) and 19% (p = 0.064) on treatment with 5 and 50 μM lunasin, respectively, whereas lunasin treatments at doses 25 and 50 μM significantly decreased PAI-1 level by 14 and 16% and leptin level by 38 and 56%, respectively (p < 0.05), in 3T3-L1 cells, showing a dose-dependence (Fig 5B and 5C). In the RAW-CM stimulation model, PAI-1 adipokine secretion by the cells treated with 25 and 50 μM lunasin significantly decreased by 19 and 20%, respectively (p < 0.05), in a dose-dependent manner (Fig 5B). In the TNF-α stimulation model, treatment with 25 and 50 μM lunasin decreased leptin secretion by 18% (p = 0.051) and 26% (p = 0.007), respectively, compared to that in the control (Fig 5C). In contrast, adiponectin secretion increased by 15% after 50 μM lunasin treatment by LPS stimulation, although the difference was not significant (Fig 5D). The contrasting roles of leptin and adiponectin were indicated in the inflammatory reaction, as leptin promoted the inflammatory process, whereas adiponectin was involved in the suppression of inflammation. This suggested that lunasin might play a significant anti-inflammatory role in adipocytes.

## Discussion

Obesity is a chronic over-nutrition disease with lipid accumulation that results in low-grade chronic inflammation in the microenvironment of adipose tissue. Accumulating evidence has revealed that various immune modifications, and pro-inflammatory cytokine overproduction around the adipose tissue resulting in the inflammatory state, are associated with adiposity [2]. This kind of inflammatory condition has been observed in the development of obesity-related diseases [4–6, 19]. This study proposed that lunasin is a promising agent with anti-inflammatory property in the obesity-related *in vitro* models. The obesity-related inflammatory models of macrophage were built using leptin, Ad-CM, and a co-culture system. The results suggested that lunasin works as a potential natural agent with anti-inflammatory property in both macrophages and adipocytes, and disrupts the crosstalk between the two cells.

Leptin was first discovered owing to its role in satiety and energy homeostasis [20]. It was found that leptin level was higher in the serum of obese subjects with leptin-resistant expression compared to that of lean subjects [18]. In the past decade, multiple functions of leptin in the immune system have been revealed [21]. Leptin works as an inflammatory mediator and can activate both adaptive and innate immunities [22]. Therefore, leptin was used as an activator in the present study to investigate the physiological conditions in obesity. Although high levels of leptin are observed in obese subjects, it could not induce apparent activation of macrophages in our study. To trigger a strong inflammatory reaction, LPS was added to the leptin model, after which the anti-inflammatory effects of lunasin became apparent in lunasin-treated RAW264.7 cells.

Studies suggest that inflammasome activation, through IL-1β activation, may contribute to insulin resistance and type II diabetes [23]. IL-1β, secreted by monocytes, macrophages, and adipocytes, is a pro-inflammatory cytokine that plays an important role in the destruction of pancreatic β-cells [24]. Many studies have shown that IL-1β blockers, such as anti-IL-1β monoclonal antibody, specifically block the inflammatory signaling cascade, which provides an effective treatment for obesity-related inflammation, insulin resistance, type II diabetes, and autoimmune diseases, like type I diabetes [24, 25]. Hence, lunasin decreased IL-1β secretion after leptin activation alone, indicating that this suppression might reduce obesity-induced inflammation and metabolic complications.

Ad-CM contains inflammatory mediators secreted by adipocytes, and it has been used to mimic the physiology of obesity [17]. This medium containing a series of mediators secreted by adipocytes is thought to be closer to the physiology of obesity than the medium with leptin supplement alone. When the RAW264.7 cells were cultured in Ad-CM, cytokine MCP-1 and IL-1β levels were decreased after lunasin treatment. In a typical physiology, macrophages play an important role in the host defense and proper development of tissue. Several cytokines produced by macrophages directly act on the inflammatory processes, participating in pathogen clearance, sensing of tissue damage, and maintenance of tissue homeostasis [26].

Stimulators, such as LPS, trigger cell active signaling through toll-like receptors (TLRs) leading to infiltration and activation of macrophages involved in the innate response [2, 27]. LPS activation initiates a cascade signaling pathway of inflammatory reactions and induces oxidative stress through recognition TLR4 present on immune cells and other types of cells, such as adipocytes [28, 29]. In obesity, LPS is derived from excess nutrients, such as saturated/free fatty acids, and gut endotoxins to activate TLR4 and perhaps other TLRs, resulting in a significant inflammatory reaction and adipocyte dysfunction [30]. Mechanistically, the obesity-related inflammation includes mTOR, JAK, JNK, IKKβ, and PI3K/Akt signaling pathways involved in inflammation activation [3, 31], which contribute to increased secretion of cytokines, such as TNF-α, IL-6, and resistin, and decreased secretion of adiponectin [30]. Moreover, gut microbiota-derived endotoxins induce pro-inflammatory and pro-oxidant reactions and subsequently cause metabolic disorders in obesity mice models [29], suggesting that adiposity is associated with progression of various metabolic complications. Recently, Achek and co-workers [32] highlighted that several agents targeting TLR are in their developmental stages or clinical trials for related immune responses and inflammatory diseases, suggesting that LPS could be a considerable stimulator to study obesity-related inflammatory response. Pro-inflammatory cytokines were inhibited by lunasin in both Ad-CM and LPS stimulated models, reflecting that lunasin can be a promising agent might possibly by disrupting the TLR signaling.

In the past decade, the intricate interaction between obesity and immune regulation has attracted the attention of various scientists. In adiposity, excess secretion of various pro-inflammatory cytokines, chemokines, and proteases, such as TNF-α, IL-6, MCP-1, leptin, and PAI-1, by infiltrating macrophages and T cells around the adipose tissue, leads to endothelial dysfunction, oxidative stress, and inflammation activation [19, 33].

MCP-1, also known as (C-C) motif ligand 2, is a member of the chemokine superfamily that functions in the recruitment and activation of monocytes during inflammation. MCP-1 has been shown to hasten macrophage infiltration in both adiposity and cancer development [34, 35]. In addition to high levels of MCP-1, hypertrophied adipocytes also express MCP-1 receptor C-C motif chemokine receptor 2 [2]. TNF-α is a conductor cytokine, whose increased expression mediates the pathogenic process of various inflammatory diseases. It has been demonstrated that blocking TNF-α and its receptor in animal models leads to resistance to development of obesity-induced insulin resistance and it may have related benefits [25]. IL-6 is another cytokine, similar to TNF-α, overexpressed in the adipose tissue in obesity [36]. These data support that lower levels of MCP-1, IL-6, TNF-α, and IL-1β induced by lunasin might help to suppress the immune activation in obesity-induced inflammatory diseases.

**Fig 6:**
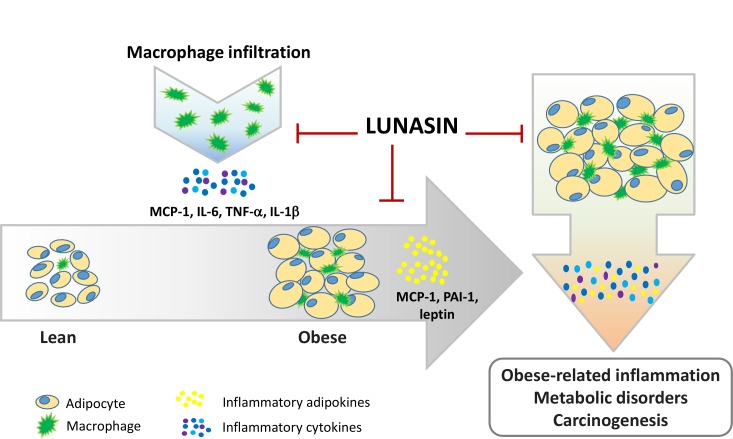
A schema of the possible influential insights on anti-inflammatory property of lunasin. In RAW264.7 macrophages, lunasin decreased inflammatory cytokine MCP-1, IL-6, TNF-α, and IL-1β productions. In 3T3-L1 adipocytes, lunasin inhibited the secretion of inflammatory adipokines MCP-1, PAI-1, and leptin. In addition, treatment with lunasin reduced the levels of these pro-inflammatory mediators, thereby disrupting the crosstalk between the two cells in a co-culture. Lunasin apparently exerted anti-inflammatory ability, possibly diminishing the obesity-induced inflammatory diseases.

Based on various researches, it has been proposed that adipose tissue dysfunction is associated with cardiovascular inflammation, insulin resistance, metabolic disorders, and carcinogenesis. Adipose tissue dysfunction in subjects with nascent metabolic syndromes leads to higher secretion of adipokines, such as IL-1, IL-6, IL-8, leptin, MCP-1, PAI-1, C-reactive protein, and serum amyloid A, by subcutaneous adipose tissue [37]. In the present study, lunasin significantly decreased MCP-1, leptin, and PAI-1 productions in 3T3-L1 adipocytes, contributing to inhibition of inflammatory mediators in this microenvironment. Leptin and adiponectin are two major adipokines secreted by adipocytes. Besides energy regulation, leptin has immune system-related roles involving immune regulation, and inflammatory response, which contribute to metabolic disorders and neoplastic cell growth [38]. The possible molecular mechanism involves generation of reactive oxygen species and expression of cyclooxygenase-2, which may trigger release of pro-inflammatory mediators through NF-κB signaling cascade [39]. In contrast, adiponectin is a negatively-regulated adipokine that is inversely associated with adiposity and inflammation. PAI-1, a serine protease inhibitor, is secreted by various cell types, including adipocytes, stromal cells, and endothelial cells, and has been associated with many pathological conditions, such as aging, cardiovascular diseases, type II diabetes, obesity, and inflammation [37]. PAI-1 shows higher expression in obese subjects and participates in adipocyte differentiation [40]. In the present study, the adiponectin level was not affected by lunasin treatment. It has demonstrated that LPS stimulation significantly reduced adiponectin and its receptor genes expression at 4 h treatment, but not at 24 h in 3T3-L1 cells [41], suggested that 24 h activation in 3T3-L1 adipocytes was too long for this mediator’s analysis. In addition, it is not always associated between adiponectin and inflammation. Onat et al. have reported the level of serum adiponectin is not significant related with inflammatory markers in Turkish adults [42]. Lunasin, a natural seed peptide, exerts many biological activities, such as anti-oxidative, anti-inflammatory, and anti-carcinogenic activities, thereby contributing to health improvement and disease prevention [10]. Currently, lunasin is being sold as a branded ingredient for promoting health, and in the past few years, its anti-cholesterol ability was revealed [43, 44]. Considering the anti-inflammatory property of lunasin, it has been shown to decrease pro-inflammatory cytokines in activated RAW264.7 cells [12], possibly through inhibition of NF-κB signaling in cardiovascular patients [13, 14], suppression of Akt phosphorylation and p65 protein expression in activated THP-1 cells [15]. Recently, the possible molecular mechanism of lunasin has demonstrated to decrease the phosphorylation of FAK, Src, Akt, and ERK, and to inactivate the nucleus translocation of NF-κB in human breast cancer cells [45]. Moreover, lunasin inhibits inflammatory response of macrophages via intervening in endocytosis-mediated integrin signaling, increased caveolin-1 expression, and internalization by macrophages [16]. In the culture model of rheumatoid arthritis, treatment with lunasin reduced cytokine production by IL-6, IL-8, and matrix metalloproteinase-3 and suppressed NF-κB activation in synovial fibroblasts [46]. In asthma mice, lunasin effectively boosted the anti-allergy immunotherapy [11]. Recently, lunasin has been reported to suppress breast cancer cell migration in the obesity-related conditional models, indicating it blocks adipocyte-cancer cell cross-talk [47]. Based on these findings, lunasin could be used as a promising natural agent to prevent and/or treat obesity-related inflammation and disease without additional side effects. A schema proffered for the possible influential insights on lunasin in the present study is depicted (Fig 6).

Taken together, the process of obesity-related inflammation is dependent on the production, performance, and crosstalk among a set of chemokines, cytokines, and inflammatory mediators [48]. This study indicated that lunasin is not only effective against inflammatory response of RAW264.7 macrophages, but also highlights this suppressive property on 3T3-L1 adipocytes, and disrupts the crosstalk between macrophages and adipocytes, particularly by inhibiting secretion of pro-inflammatory mediators, might benefit to ameliorate obesity-induced inflammatory diseases. In the future, the *in vivo* animal study should be conducted to confirm the effect and safety of lunasin. New genomic and proteomic approaches, novel techniques, and cooperation among researchers are need to pursuit comprehensive understanding.

## Supporting information

S1 Appendix Raw data. All relevant raw data are within this supporting information file.
(PDF)
