# Supplementary material for: Lunasin attenuates obesity-related inflammation in RAW264.7 cells and 3T3-L1 adipocytes by inhibiting inflammatory cytokine production
Source: PLoS One. 2017 Feb 9;12(2):e0171969. doi: 10.1371/journal.pone.0171969 (PMC5300240; doi:10.1371/journal.pone.0171969)
Supplement: S1 Appendix — All relevant raw data are within this supporting information file. (PDF) [file pone.0171969.s001.pdf]

Figure 1. Experimental design and basal conditions

B. lunasin_cell viability

lunasin(uM)	test 1		test 2		test 3						LSD_p value
	OD	viability%	OD	viability%	OD	viability%	mean of viability%	SD	n	SEM	viability%
control	0.655	100.0	0.628	100.0	0.677	100.0	100.0	0.0	3	0.0	
0.2	0.632	107.8	0.658	99.7	0.743	98.0	101.9	5.2	3	3.0	0.780
1	0.63	100.0	0.761	100.0	0.711	100.0	100.0	0.0	3	0.0	1.000
10	0.693	90.9	0.764	99.6	0.666	106.8	99.1	7.9	3	4.6	0.891
50	0.747	91.2	0.762	86.1	0.653	111.6	96.3	13.5	3	7.8	0.575
200	0.739	94.5	0.725	82.2	0.419	101.7	92.8	9.8	3	5.7	0.284

C. LPS_cell viability

LPS dose(ng/ml)	test 1			test 2			test 3							
	mean	-blank	viability%	mean	-blank	viability%	Mean	-blank	viability%	mean of viability%	SD	n	SEM	LSD_p value
blank	0.055			0.053			0.05							
0	2.939	2.884	100.0	0.847	0.794	100.0	2.387	2.337	100.0	100.0	0.0	3	0.0	
10	3.373	3.318	115.1	1.025	0.972	122.5	2.762	2.712	116.0	117.9	4.0	3	2.3	0.157
25	3.322	3.267	113.3	1.132	1.079	135.9	2.744	2.694	115.3	121.5	12.5	3	7.2	0.093
50	3.185	3.130	108.5	1.146	1.093	137.7	2.884	2.834	121.3	122.5	14.6	3	8.4	0.080
100	3.232	3.177	110.2	1.205	1.152	145.1	2.707	2.657	113.7	123.0	19.3	3	11.1	0.074
250	2.938	2.883	100.0	1.095	1.042	131.3	2.786	2.736	117.1	116.1	15.7	3	9.1	0.199
500	2.654	2.599	90.1	1.047	0.994	125.2	2.604	2.554	109.3	108.2	17.5	3	10.1	0.506
1000	2.723	2.668	92.5	1.074	1.021	128.6	2.222	2.172	92.9	104.7	20.7	3	12.0	0.704

D. LPS_MCP-1

LPS (ng/mL)	pg/ml		% of control						
test	1	2	1	2	mean	SD	n	SEM	LSD_p value
0	23.4	7.3	100.0	100.0	100	0	2	0	
10	497.4	114.6	2122.2	1559.0	1840.6	398.2	2	281.6	0.062
25	596.4	251.9	2544.4	3427.9	2986.2	624.7	2	441.7	0.007
50	1023.4	384.5	4366.7	5231.1	4798.9	611.3	2	432.2	0.000
100	2200.5	684.5	9388.9	9313.1	9351.0	53.6	2	37.9	0.000
250	3257.8	990.5	13900.0	13477.0	13688.5	299.1	2	211.5	0.000
500	3862.0	1001.3	16477.8	13624.6	15051.2	2017.5	2	1426.6	0.000
1000	3502.6	1070.0	14944.4	14559.0	14751.7	272.5	2	192.7	0.000

Figure 2. Lunasin inhibited inflammatory cytokine production in leptin-conditioned RAW264.7 cells

A. leptin dose_cell viability

leptin														
leptin dose (ng/mL)	test 1			test 2			test 3			viability%				
	Mean	-blank	viability%	mean	-blank	viability%	mean	-blank	viability%	mean of viability%	SD	n	SEM	LSD_p value
blank	0.055			0.054			0.05							
0	1.725	1.670	100.0	0.728	0.674	100.0	1.992	1.942	100.0	100.0	0.0	3	0.0	
50	2.099	2.044	121.7	0.878	0.824	122.3	1.910	1.860	95.8	113.3	15.2	3	8.7	0.171
100	1.976	1.921	114.6	0.817	0.763	113.3	2.021	1.971	101.5	109.8	7.2	3	4.2	0.304
200	2.113	2.058	122.5	0.912	0.858	127.3	1.997	1.947	100.2	116.7	14.5	3	8.3	0.091
400	2.116	2.061	122.7	0.860	0.806	119.6	2.045	1.995	102.7	115.0	10.7	3	6.2	0.125
800	2.109	2.054	122.3	0.902	0.848	125.8	2.067	2.017	103.9	117.3	11.8	3	6.8	0.080
1600	2.093	2.038	121.4	0.901	0.847	125.7	2.056	2.006	103.3	116.8	11.9	3	6.9	0.089

leptin+LPS														
leptin dose (ng/mL)	test 1			test 2			test 3			viability%				
	Mean	-blank	viability%	mean	-blank	viability%	mean	-blank	viability%	mean of viability%	SD	n	SEM	LSD_p value
blank	0.055			0.054			0.05							
0	2.463	2.408	100.0	0.858	0.804	100.0	2.334	2.284	100.0	100.0	0.0	3	0.0	
50	2.775	2.720	112.7	1.015	0.961	119.5	2.457	2.407	105.4	112.5	7.0	3	4.1	0.125
100	2.525	2.470	102.5	1.066	1.012	125.8	2.548	2.498	109.4	112.6	12.0	3	6.9	0.124
200	2.536	2.481	103.0	1.042	0.988	122.9	2.636	2.586	113.2	113.0	10.0	3	5.8	0.112
400	2.739	2.684	111.2	1.066	1.012	125.8	2.499	2.449	107.2	114.8	9.8	3	5.7	0.076
800	2.283	2.228	92.7	1.005	0.951	118.3	2.527	2.477	108.5	106.5	12.9	3	7.4	0.412
1600	2.692	2.637	109.3	1.020	0.966	120.2	2.438	2.388	104.5	111.4	8.0	3	4.6	0.162

B. leptin dose_MCP-1

leptin																
leptin dose (ng/mL)	pg/ml								% of control							
test	1	2	3	4	mean	SD	n	SEM	1	2	3	mean	SD	n	SEM	LSD_p value
0	13.8	14.0	2.2	0.7	7.7	7.2	4	3.6	100	100	100.0	100.0	0.0	3	0.0	
50	12.3	10.9	2.7	0.7	6.6	5.8	4	2.9	89	122	102.2	104.3	16.2	3	9.4	0.850
100	13.3	14.4	3.4	0.8	8.0	6.9	4	3.5	96	152	117.4	121.9	27.9	3	16.1	0.350
200	12.3	12.4	3.7	0.8	7.3	5.9	4	3.0	89	169	123.9	127.4	39.9	3	23.1	0.245
400	14.0	9.0	3.6	0.9	6.9	5.8	4	2.9	101	165	134.8	133.5	31.8	3	18.3	0.159
800	15.6	12.7	3.4	1.0	8.2	7.1	4	3.5	113	156	152.2	140.4	23.8	3	13.7	0.095
1600	15.1	11.0	3.8	1.1	7.7	6.4	4	3.2	110	173	160.9	147.9	33.8	3	19.5	0.052
leptin+LPS																
leptin dose (ng/mL)	pg/ml								% of control							
test	1	2	3	4	mean	SD	n	SEM	1	2	3	mean	SD	n	SEM	LSD_p value
0	1059.7	41.2	17.0	80.3	299.5	507.4	4	253.7	100.0	100.0	100.0	100.0	0.0	3	0.0	

50	1636.7	69.6	20.1	105.1	457.9	786.7	4	393.3	168.9	118.5	130.9	139.4	26.3	3	15.2	0.136
100	1682.6	76.8	17.7	103.8	470.2	809.1	4	404.5	186.5	104.5	129.2	140.1	42.1	3	24.3	0.130
200	1944.9	82.5	22.8	105.7	539.0	938.0	4	469.0	200.4	134.2	131.6	155.4	39.0	3	22.5	0.043
400	2066.2	76.4	19.5	120.3	570.6	997.9	4	499.0	185.6	115.2	149.8	150.2	35.2	3	20.3	0.064
800	1312.1	76.0	21.7	108.8	379.7	622.7	4	311.3	184.6	128.1	135.6	149.4	30.7	3	17.7	0.067
1600	2263.0	43.7	24.3	95.7	606.6	1104.6	4	552.3	106.0	143.2	119.1	122.8	18.9	3	10.9	0.376

C. leptin_MCP-1

MCP-1 (pg/mL) lunasin(uM)		pg/ml								% of control								
		test 1	test 2	test 3	test 4	mean	SD	n	SEM	test 1	test 2	test 3	test 4	mean	SD	n	SEM	LSD_p value
leptin 200ng/ml	0	36.3	19.6	11.6	22.9	22.6	10.3	4	5.1	100.0	100.0	100.0	100.0	100.0	0.0	4	0.0	
	1	28.0	19.1	13.0		20.1	7.5	3	4.4	77.2	97.6	112.1		95.6	17.5	3	10.1	0.835
	10	26.5	16.0	13.0	32.7	22.1	9.2	4	4.6	72.9	81.7	112.1	143.0	102.4	31.9	4	15.9	0.901
	50	36.3	15.5	13.3	36.6	25.5	12.8	4	6.4	100.0	79.3	114.8	160.1	113.5	34.3	4	17.1	0.489
LPS 100ng/ml	0	3130.7	4608.0	5835.2	5676.1	4812.5	1246.6	4	623.3	100.0	100.0	100.0	100.0	100.0	0.0	4	0.0	
	1	2448.9	4914.8	4943.2	4323.9	4157.7	1174.4	4	587.2	78.2	106.7	84.7	76.2	86.4	14.0	4	7.0	0.039
	10	2039.8	3215.9	4233.0	3664.8	3288.4	930.6	4	465.3	65.2	69.8	72.5	64.6	68.0	3.8	4	1.9	0.000
	50	1840.9	2068.2	3460.2	2818.2	2546.9	738.3	4	369.2	58.8	44.9	59.3	49.6	53.2	7.1	4	3.5	0.000
leptin+ LPS	0	2573.9	4846.6	6409.1	6250.0	5019.9	1775.4	4	887.7	100.0	100.0	100.0	100.0	100.0	0.0	4	0.0	
	1	2193.2	3977.3	4278.4	4460.2	3727.3	1041.9	4	521.0	85.2	82.1	66.8	71.4	76.3	8.7	4	4.4	0.001
	10	1738.6	3448.9	3403.4	3693.2	3071.0	897.3	4	448.7	67.5	71.2	53.1	59.1	62.7	8.2	4	4.1	0.000
	50	1511.4	2715.9	2983.0	2840.9	2512.8	676.5	4	338.2	58.7	56.0	46.5	45.5	51.7	6.7	4	3.3	0.000

D. leptin_IL-6

IL-6 (pg/mL)	lunasin(uM	pg/ml								% of control								
		test 1	test 2	test 3	test 4	mean	SD	n	SEM	test 1	test 2	test 3	test 4	mean	SD	n	SEM	LSD_p value
leptin 200ng/ml	0	15.9	7.4	9.9	8.9	10.5	3.7	4	1.9	100.0	100.0	100.0	100.0	100.0	0.0	4	0.0	

	1	4.9	9.4	6.9	9.4	7.6	2.2	4	1.1	30.6	127.2	69.5	105.6	83.3	42.4	4	21.2	0.443
	10	7.4	7.9	7.9	10.4	8.4	1.4	4	0.7	46.4	106.8	79.7	116.9	87.5	31.6	4	15.8	0.564
	50	6.9	5.3	6.9	6.9	6.5	0.8	4	0.4	43.2	72.8	69.5	77.4	65.7	15.4	4	7.7	0.128
LPS 100ng/ml	0	71.7	221.7	159.4	184.7	159.4	63.8	4	31.9	100.0	100.0	100.0	100.0	100.0	0.0	4	0.0	
	1	55.7	210.3	135.2	139.7	135.2	63.2	4	31.6	77.7	94.8	88.4	75.6	84.1	9.1	4	4.5	0.087
	10	63.7	227.4	136.6	118.7	136.6	68.0	4	34.0	88.8	102.6	90.5	64.3	86.5	16.1	4	8.0	0.141
	50	61.7	178.9	115.1	104.7	115.1	48.4	4	24.2	86.1	80.7	83.4	56.7	76.7	13.5	4	6.8	0.017
leptin+ LPS	0	90.7	255.7	172.0	169.7	172.0	67.4	4	33.7	100.0	100.0	100.0	100.0	100.0	0.0	4	0.0	
	1	59.7	211.7	145.0	163.7	145.0	63.4	4	31.7	65.8	82.8	80.4	96.5	81.4	12.6	4	6.3	0.010
	10	69.7	218.7	146.0	149.7	146.0	60.9	4	30.4	76.8	85.5	78.9	88.2	82.4	5.4	4	2.7	0.013
	50	66.7	194.7	125.0	113.7	125.0	52.9	4	26.4	73.5	76.1	70.3	67.0	71.7	4.0	4	2.0	0.000

E. leptin_TNF-α

TNF- α (pg/mL)	lunasin(uM	pg/ml								% of control								
		test 1	test 2	test 3	test 4	mean	SD	n	SEM	test 1	test 2	test 3	test 4	mean	SD	n	SEM	LSD_p value
leptin 200ng/ml	0	64.7	63.7	53.7	72.7	63.7	7.8	4	3.9	100.0	100.0	100.0	100.0	100.0	0.0	4	0.0	
	1	51.7	95.7	54.7	72.7	68.7	20.2	4	10.1	79.9	150.2	101.9	100.0	108.0	29.9	4	14.9	0.848
	10	98.7	53.7	99.7	137.7	97.5	34.4	4	17.2	152.6	84.3	185.7	189.4	153.0	48.7	4	24.3	0.217
	50	242.7	401.7			369.0	89.9	4	45.0	375.1			624.1	499.6	176.0	2	124.5	0.000
LPS 100ng/ml	0	20342.5	17517.5	18767.5	26842.5	20867.5	4147.6	4	2073.8	100.0	100.0	100.0	100.0	100.0	0.0	4	0.0	
	1	18242.5	17167.5	15867.5	21992.5	18317.5	2635.4	4	1317.7	89.7	98.0	84.5	81.9	88.5	7.1	4	3.5	0.247
	10	16192.5	17817.5	15717.5	15867.5	16398.8	966.4	4	483.2	79.6	101.7	83.7	59.1	81.0	17.5	4	8.7	0.065
	50	18467.5	17567.5	15742.5	16517.5	17073.8	1192.8	4	596.4	90.8	100.3	83.9	61.5	84.1	16.5	4	8.2	0.116
leptin+ LPS	0	20342.5	19167.5	19467.5	19492.5	19617.5	505.4	4	252.7	100.0	100.0	100.0	100.0	100.0	0.0	4	0.0	
	1	17217.5	18967.5	17467.5	14867.5	17130	1694.8	4	847.4	84.6	99.0	89.7	76.3	87.4	9.5	4	4.7	0.035
	10	17467.5	16467.5	15917.5		16617.5	785.9	3	453.7	85.9	85.9	81.8		84.5	2.4	3	1.4	0.133

	50	17542.5	17167.5	16067.5	16342.5	16780	690.6	4	345.3	86.2	89.6	82.5	83.8	85.5	3.1	4	1.5	0.018

F. leptin_IL-1β

IL-1 β (pg/mL)	lunasin(uM	pg/ml								% of control								
		test 1	test 2	test 3	test 4	mean	SD	n	SEM	test 1	test 2	test 3	test 4	mean	SD	n	SEM	LSD_p value
leptin 200ng/ml	0	10.8	10.7	10.3	9.5	10.3	0.6	4	0.3	100.0	100.0	100.0	100.0	100.0	0.0	4	0.0	
	1	10.5	10.2	9.3	9.5	9.9	0.6	4	0.3	96.9	95.3	90.3	100.0	95.6	4.0	4	2.0	0.134
	10	10.5	10.7	9.7	9.3	10.0	0.6	4	0.3	96.9	100.0	93.5	98.2	97.2	2.7	4	1.4	0.318
	50	10.7	9.7	9.8	8.2	9.6	1.0	4	0.5	98.5	90.6	95.2	86.0	92.6	5.4	4	2.7	0.017
LPS 100ng/ml	0	78.2	98.2	76.4	101.8	88.6	13.2	4	6.6	100.0	100.0	100.0	100.0	100.0	0.0	4	0.0	
	1	100.9	102.7	64.5	108.2	94.1	19.9	4	10.0	129.1	104.6	84.5	106.3	106.1	18.2	4	9.1	0.645
	10	83.6	92.7	69.1	108.2	88.4	16.4	4	8.2	107.0	94.4	90.5	106.3	99.5	8.3	4	4.2	0.837
	50	74.5	85.5	60.0	90.9	77.7	13.6	4	6.8	95.3	87.0	78.6	89.3	87.6	6.9	4	3.5	0.332
leptin+ LPS	0		93.6	84.5	117.3	98.5	16.9	3	9.8		100.0	100.0	100.0	100.0	0.0	3	0.0	
	1		102.7	70.0	96.4	89.7	17.4	3	10.0		109.7	82.8	82.2	91.6	15.7	3	9.1	0.378
	10		84.5	70.9	122.7	92.7	26.9	3	15.5		90.3	83.9	104.7	92.9	10.6	3	6.1	0.460
	50		73.6	60.0	90.9	74.8	15.5	3	8.9		78.6	71.0	77.5	75.7	4.1	3	2.4	0.024

Figure 3. Lunasin inhibited inflammatory cytokine production in Ad-CM-conditioned RAW264.7 cells

A. Ad-CM %_cell viability

Ad-CM																	
Ad-CM	test 1			test 2			test 3			test 4			viability%			
% of CM	mean	-blank	viability%	mean	-blank	viability%	mean	-blank	viability%	mean	-blank	viability%	mean of viability%	SD	n SEM	LSD_p value
blank	0.055			0.052			0.054			0.05						
0	1.826	1.771	100.0	1.271	1.219	100.0	0.836	0.782	100.0	1.829	1.779	100.0	100.0	0.0	0.0	
10	1.931	1.876	105.8	1.706	1.654	134.2	0.857	0.803	102.7	1.804	1.754	98.6	110.3	16.2	8.1	0.230
25	2.113	2.058	115.7	1.517	1.465	119.3	0.926	0.872	111.5	1.712	1.662	93.4	110.0	11.5	5.7	0.245
50				1.333	1.281	104.9	0.819	0.765	97.7	1.513	1.463	82.3	95.0	11.6	6.7	0.577
Ad-CM+LPS 100 ng/mL																
Ad-CM	test 1			test 2			test 3			test 4			viability%			
% of CM	mean	-blank	viability%	mean	-blank	viability%	mean	-blank	viability%	mean	-blank	viability%	mean of viability%	SD	n SEM	LSD_p value
blank	0.055			0.052			0.054			0.05						
0	2.382	2.327	100	1.393	1.341	100.0	0.976	0.922	100.0	2.426	2.376	100.0	100.0	0.0	0	
10	2.457	2.402	103.1	1.343	1.291	96.4	1.127	1.073	116.4	2.263	2.213	93.2	102.3	10.3	5.1	0.838
25	2.365	2.310	99.3	1.954	1.902	140.3	0.961	0.907	98.3	2.056	2.006	84.424	105.6	24.1	12.1	0.618
50				1.259	1.207	90.4	1.019	0.965	104.6	1.760	1.710	71.964	89.0	16.4	9.4	0.369

B. Ad-CM %_MCP-1

Ad-CM													
Ad-CM	pg/ml						% of control						
% of CM	1	2	mean	SD	n	SEM	1	2	mean	SD	n	SEM	LSD_p value
0	3.1	4.6	3.8	1.1	2	0.8	100	100	100.0	0.0	2	0.0	
10	130.8	148.3	139.6	12.4	2	8.8	4289	3259	3773.9	727.7	2	514.6	0.022
25	196.3	220.8	208.6	17.3	2	12.3	6436	4853	5644.4	1119.6	2	791.7	0.005
50	250.8	277.3	264.1	18.7	2	13.3	8223	6095	7158.7	1505.0	2	1064.2	0.002

Ad-CM+LPS 100 ng/mL													
Ad-CM	pg/ml						% of control						
% of CM	1	2	mean	SD	n	SEM	1	2	mean	SD	n	SEM	LSD_p value
0	138.0	48.0	93.0	63.6	2	45.0	100.0	100.0	100.0	0.0	2	0.0	
10	263.0	293.0	278.0	21.2	2	15.0	190.6	610.4	400.5	296.9	2	209.9	0.661
25	533.0	615.5	574.3	58.3	2	41.3	386.2	1282.3	834.3	633.6	2	448.0	0.312
50	943.0	1048.0	995.5	74.2	2	52.5	683.3	2183.3	1433.3	1060.7	2	750.0	0.104

C. Ad-CM_MCP-1

MCP-1 (pg/mL) lunasin(uM		pg/ml								% of control								
		test 1	test 2	test 3	test 4	mean	SD	n	SEM	test 1	test 2	test 3	test 4	mean	SD	n	SEM	LSD_p value
Ad-CM	0	464.0	528.3	403.3	467.1	442.1	68.8	4	34.4	100.0	100.0	100.0	100.0	100.0	0.0	4	0.0	
	1	411.3	422.1	398.8	316.2	369.9	82.2	4	41.1	88.6	79.9	98.9	67.7	83.8	13.2	4	6.6	0.016
	10	426.5	448.8	374.7	370.8	385.7	69.3	4	34.6	91.9	85.0	92.9	79.4	87.3	6.4	4	3.2	0.047
	50	393.5	388.1		363.4	356.1	60.2	3	34.7	84.8	73.5		77.8	78.7	8.0	3	4.6	0.005
LPS 100ng/ml	0	1146.2	2174.2	1524.2	4872.2	2429.2	1683.1	4	841.5	100.0	100.0	100.0	100	100.0	0.0	4	0.0	
	1	1022.2	2064.2	1440.2	4816.7	2335.8	1708.4	4	854.2	89.2	94.9	94.5	98.9	94.4	4.0	4	2.0	0.385
	10	1124.2	1586.2	1204.2	4879.6	2198.6	1798.7	4	899.3	98.1	73.0	79.0	100.2	87.5	13.6	4	6.8	0.070
	50	1040.2	1490.2	1376.2	4201.9	2027.1	1462.4	4	731.2	90.8	68.5	90.3	86.2	83.9	10.5	4	5.2	0.024
Ad-CM+ LPS	0	712.2	1396.2	1342.2	4357.4	1952.0	1633.4	4	816.7	100.0	100.0	100.0	100.0	100.0	0.0	4	0.0	
	1	686.2	1474.2	992.2	3538.9	1672.9	1285.6	4	642.8	96.3	105.6	73.9	81.2	89.3	14.3	4	7.2	0.343
	10	702.2	1104.2	1076.2	2964.8	1461.9	1018.6	4	509.3	98.6	79.1	80.2	68.0	81.5	12.7	4	6.3	0.129
	50	588.2	1086.2	1110.2	3264.8	1512.4	1192.8	4	596.4	82.6	77.8	82.7	74.9	79.5	3.8	4	1.9	0.017

D. Ad-CM_IL-6

IL-6 (pg/mL) lunasin(uM	pg/ml								% of control								
	test 1	test 2	test 3	test 4	mean	SD	n	SEM	test 1	test 2	test 3	test 4	mean	SD	n	SEM	LSD_p value

Ad-CM 200ng/ml	0	12.8	9.3	9.7	12.4	11.0	1.8	4	0.9	100.0	100.0	100.0	100.0	100.0	0.0	4	0.0	
	1	10.5	9.7	10.1	8.9	9.8	0.7	4	0.3	81.9	104.1	104.0	72.0	90.5	16.1	4	8.1	0.444
	10	14.3	8.9	9.3	9.3	10.5	2.6	4	1.3	112.0	95.9	96.0	75.2	94.8	15.1	4	7.6	0.671
	50	15.1	8.9		8.9	11.0	3.6	3	2.1	118.1	95.9		72.0	95.3	23.0	3	13.3	0.725
LPS 100ng/ml	0		420.6	1038.3	1016.9	825.3	350.6	3	202		100.0	100.0	100.0	100.0	0.0	3	0.0	
	1		323.4	859.6	704.9	629.3	276.0	3	159		76.9	82.8	69.3	76.3	6.8	3	3.9	0.093
	10		254.9	799.6	622.3	558.9	277.8	3	160		60.6	77.0	61.2	66.3	9.3	3	5.4	0.013
	50		283.9	828.9	574.3	581.3	244.3	3	141		67.5	79.8	56.5	67.9	11.7	3	6.7	0.001
Ad-CM+ LPS	0	94.8	153.4	744.7	196.3	297.3	301.1	4	151	100.0	100.0	100.0	100.0	100.0	0.0	4	0.0	
	1	86.3	126.3	700.7		304.4	343.7	3	160	91.1	82.3	94.1		89.2	6.1	3	3.5	0.226
	10	92.5	86.3	583.3	160.6	230.7	237.5	4	119	97.6	56.2	78.3	81.8	78.5	17.0	4	8.5	0.017
	50	83.2	149.1	470.0	134.9	209.3	176.1	4	88	87.8	97.2	63.1	68.7	79.2	16.0	4	8.0	0.020

E. Ad-CM_TNF-α

TNF-α (pg/mL) lunasin(uM		pg/ml							% of control							
		test 1	test 2	test 3	mean	SD	n	SEM	test 1	test 2	test 3	mean	SD	n	SEM	LSD_p value
Ad-CM 200ng/ml	0	457.2	272.0	346.3	358.5	93.2	3	53.8	100.0	100.0	100.0	100.0	0.0	3	0.0	
	1	117.2	239.4	374.5	243.7	128.7	3	74.3	25.6	88.0	108.1	73.9	43.0	3	24.8	0.363
	10	275.4	313.4	369.9	319.6	47.6	3	27.5	60.2	115.2	106.8	94.1	29.6	3	17.1	0.808
	50	114.5		379.9	247.2	187.7	2	132.7	25.0		109.7	67.4	59.9	2	42.3	0.345
LPS 100ng/ml	0	11907.3	14089.1	12525.5	12840.6	1124.5	3	649.3	100.0	100.0	100.0	100.0	0.0	3	0.0	
	1	8089.1	10070.9	10161.8	9440.6	1171.3	3	676.3	67.9	71.5	81.1	73.5	6.8	3	3.9	0.018
	10	6834.5	10925.5	13034.5	10264.8	3152.3	3	1820.1	57.4	77.5	104.1	79.7	23.4	3	13.5	0.024
	50	5998.2	10198.2	12380.0	9525.5	3243.7	3	1872.8	50.4	72.4	98.8	73.9	24.3	3	14.0	0.016
Ad-CM+ LPS	0	11198.2	13234.5	15107.3	13180.0	1955.1	3	1128.8	100.0	100.0	100.0	100.0	0.0	3	0.0	
	1	9325.5	10889.1	11961.8	10725.5	1325.8	3	765.5	83.3	82.3	79.2	81.6	2.1	3	1.2	0.001

		10	8034.5	11743.6	12707.3	10828.5	2467.1	3	1424.4	71.7	88.7	84.1	81.5	8.8	3	5.1	0.002
		50	7452.7	10343.6	14089.1	10628.5	3327.3	3	1921.1	66.6	78.2	93.3	79.3	13.4	3	7.7	0.000

F. Ad-CM_IL-1β

IL-1 β (pg/mL)	lunasin(uM	pg/ml								% of control								
		test 1	test 2	test 3	test 4	mean	SD	n	SEM	test 1	test 2	test 3	test 4	mean	SD	n	SEM	LSD_p value
Ad-CM 200ng/ml	0	10.0	11.2	7.1	10.0	9.6	1.8	4	0.9	100.0	100.0	100.0	100.0	100.0	0.0	4	0.0	
	1	6.6	8.6	6.7	9.1	7.8	1.3	4	0.7	65.5	76.9	95.1	91.4	82.2	13.6	4	6.8	0.015
	10	6.2	9.0	6.7	9.0	7.7	1.5	4	0.7	62.1	80.0	95.1	89.7	81.7	14.5	4	7.3	0.061
	50	7.8	8.8	7.6	8.8	8.2	0.7	4	0.3	77.6	78.5	107.3	87.9	87.8	13.8	4	6.9	0.090
LPS 100ng/ml	0	119.7	274.4	349.2	195.8	234.8	99.0	4	49.5	100.0	100.0	100.0	100.0	100.0	0.0	4	0.0	
	1	66.0	177.6	182.8	150.8	144.3	54.1	4	27.0	55.1	64.7	79.6	77.0	69.1	11.4	4	5.7	0.004
	10	70.2	160.7	241.8	200.8	168.4	73.3	4	36.7	58.7	58.6	90.5	102.6	77.6	22.4	4	11.2	0.026
	50	82.8	189.2	191.3	169.5	158.2	51.2	4	25.6	69.2	68.9	89.1	86.6	78.5	10.9	4	5.4	0.031
Ad-CM+ LPS	0	73.4	299.7	332.3	190.8	224.0	117.3	4	58.6	100.0	100.0	100.0	100.0	100.0	0.0	4	0.0	
	1	57.6	230.2	269.2	183.3	185.0	92.0	4	46.0	78.5	76.8	93.7	96.1	86.3	10.0	4	5.0	0.056
	10	67.1	259.7	293.4	203.3	205.8	99.7	4	49.9	91.4	86.7	81.1	106.6	91.4	10.9	4	5.5	0.214
	50		179.7	134.4	139.5	151.2	24.8	3	14.3		60.0	71.7	73.1	68.3	7.2	3	4.2	0.001

Figure 4. Lunasin abated inflammatory cytokine production in the RAW264.7 cell and mature 3T3-L1 adipocyte co-culture

B.

	ng/m							LSD
	test1	test2	test3	MEAN	SD	n	SEM	p value
3T3-only	0.3	0.3	0.3	0.3	0.0	3	0.0	0.3067
RAW-only	0.0	0.0	0.0	0.0	0.0	3	0.0	0.2980
cocu-control	3.1	3.2	3.8	3.4	0.4	3	0.2	
cocu+lps-control	95.9	104.6	99.3	99.9	4.4	3	2.5	0.0000

C. co-culture_cytokine

MCP-1	pg/ml							% of control							
lunasin(uM)	test 1	test 2	test 3	mean	SD	n	SEM	test 1	test 2	test 3	mean	SD	n	SEM	LSD_p value
0	95.9	104.6	99.3	99.9	4.4	3	2.5	100.0	100.0	100.0	100.0	0.0	3	0.0	
10	88.0	106.4	92.8	95.7	9.5	3	5.5	91.8	101.8	93.4	95.7	5.4	3	3.1	0.807
50	91.2	97.2	86.4	91.6	5.4	3	3.1	95.1	93.0	87.0	91.7	4.2	3	2.4	0.640

IL-6	pg/ml								% of control								
lunasin(uM)	test 1	test 2	test 3	test 4	mean	SD	n	SEM	test 1	test 2	test 3	test 4	mean	SD	n	SEM	LSD p value
0	7.06	17.3	20.6	14.9	15.0	5.8	4	2.9	100.0	100.0	100.0	100.0	100.0	0.0	4	0.0	
10	6.66	18.6	23.1	17.7	16.5	7.0	4	3.5	94.3	107.9	112.1	118.7	108.3	10.3	4	5.1	0.047
50	6.17	16.5	19.2	13.3	13.8	5.6	4	2.8	87.4	95.4	93.1	89.5	91.3	3.6	4	1.8	0.037

TNF-α	pg/ml								% of control								
lunasin(uM)	test 1	test 2	test 3	test 4	mean	SD	n	SEM	test 1	test 2	test 3	test 4	mean	SD	n	SEM	LSD_p value
0	1.9	2.7	4.1	2.6	2.9	0.92	4	0.5	100.0	100.0	100.0	100.0	100.0	0.0	4	0.0	
10	2.1	2.9	2.9	2.4	2.6	0.39	4	0.2	108.0	105.4	70.4	92.1	94.0	17.2	4	8.6	0.516
50		1.9	3.8	2.4	2.7	0.99	3	0.6		69.2	91.8	89.0	83.4	12.3	3	7.1	0.107

IL-1β	pg/ml								% of control								
lunasin(uM)	test 1	test 2	test 3	test 4	mean	SD	n	SEM	test 1	test 2	test 3	test 4	mean	SD	n	SEM	LSD_p value
0	66.5	46.5	37.1	40.9	47.8	13.1	4	6.5	100.0	100.0	100.0	100.0	100.0	0.0	4	0.0	
10	50.9	35.9	28.7	35.4	37.7	9.4	4	4.7	76.6	77.3	77.5	86.4	79.5	4.7	4	2.3	0.081
50	37.1		32.6	47.6	41.2	7.6	4	3.8	55.7		88.0	116.3	86.7	30.3	3	17.5	0.298

Fig 5_Lunasin inhibited pro-inflammatory adipokine production in 3T3-L1 adipocytes

A. MCP-1

MCP-1_DMEM1%FBS

MCP-1	pg/ml				mean	SD	SEM	n	LSD_p value	% of control				mean	SD	SEM	n	LSD_p value
test	1	2	3	4						1	2	3	4					
control	516.2	437.9	485.6	360.7	450.1	67.73	33.86	4		100.0	100.0	100.0	100.0	100.0	0.0	0.0	4	
Lunasin 5 uM	450.9	524.1	441.7	322.2	418.9	80.65	36.07	5	0.573	87.4	119.7	91.0	89.3	96.8	15.3	6.8	4	0.759
Lunasin 25 uM	393.0	525.6	466.1	313.9	419.5	80.17	35.85	5	0.581	76.1	120.0	96.0	87.0	94.8	18.7	8.4	4	0.612
Lunasin 50 uM	403.1	429.2	439.3		401.1	48.00	24.00	4	0.405	78.1	98.0	90.5		88.9	10.1	5.0	3	0.324

MCP-1_TNF-a 10 ng/ml

MCP-1	pg/ml				mean	SD	SEM	n	LSD_p value	% of control			mean	SD	SEM	n	LSD_p value
test	1	2	3	4						1	2	3					
control	89072.5	89072.5	90231.9		89458.9	669.39	386.47	3		100.0	100.0	100.0	100.0	0.0	0.0	3	
Lunasin 5 uM	75739.1	81246.4	54000.0	86753.6	74434.8	14346.12	7173.06	4	0.079	91.2	60.6	96.1	82.7	19.2	9.6	3	0.110
Lunasin 25 uM	76608.7	75739.1	87333.3	79797.1	79869.6	5272.79	2636.39	4	0.246	85.0	98.0	88.4	90.5	6.8	3.4	3	0.359
Lunasin 50 uM	57478.3	66753.6	62985.5	89652.2	69217.4	14145.60	7072.80	4	0.023	74.9	70.7	99.4	81.7	15.5	7.7	3	0.094

MCP-1_50%RAW-CM

MCP-1	pg/ml	mean	SD	SEM	n	LSD_p value	% of control	mean	SD	SEM	n	LSD_p value

test	1	2	3						1	2	3					
control	224024.4	182561.0	171097.6	192561.0	27844.4	16076	3		100.0	100.0	100.0	100.0	0.0	0.0	3	
Lunasin 5 uM	245243.9	174756.1	155975.6	191991.9	47063.9	27172	3	0.985	109.5	95.7	91.2	98.8	9.5	5.5	3	0.870
Lunasin 25 uM	235000.0	190609.8	172561.0	199390.2	32132.2	18552	3	0.820	104.9	104.4	100.9	103.4	2.2	1.3	3	0.644
Lunasin 50 uM	181341.5	194756.1	130122.0	168739.8	34110.0	19693	3	0.434	80.9	106.7	76.1	87.9	16.5	9.5	3	0.121

MCP-1_LPS 1000 ng/ml

MCP-1	pg/ml			mean	SD	SEM	n	LSD_p value	% of control			mean	SD	SEM	n	LSD_p value
test	1	2	3						1	2	3					
control	35762.2	55335.4	30152.4	40416.7	13220.9	7633	3		100.0	100.0	100.0	100.0	0.0	0.0	3	
Lunasin 5 uM	27957.3	32408.5	26493.9	28953.3	3080.5	1779	3	0.147	78.2	58.6	87.9	74.9	14.9	8.6	3	0.022
Lunasin 25 uM	33628.0	45762.2	25823.2	35071.1	10047.5	5801	3	0.481	94.0	82.7	85.6	87.4	5.9	3.4	3	0.205
Lunasin 50 uM	24359.8	39969.5	30701.2	31676.8	7850.5	4532	3	0.259	68.1	72.2	101.8	80.7	18.4	10.6	3	0.064

B. PAI-1

PAI-1_DMEM1%FBS

PAI-1	ng/ml				mean	SD	SEM	n	LSD_p value	% of control				mean	SD	SEM	n	LSD_p value
test	1	2	3	4						1	2	3	4					
control	13.8	14.9	4.1	4.0	9.2	6.6	3.3	4		100.0	100.0	100.0	100.0	100.0	0.0	0.0	4	
Lunasin 5 uM	13.6	15.9	4.5	4.6	9.7	6.7	3.4	4	0.917	98.8	107.3	110.1	114.4	107.7	6.6	3.3	4	0.310
Lunasin 25 uM	12.4	15.7	5.3	4.7	9.5	6.3	3.2	4	0.940	90.5	105.6	128.4	114.9	109.8	16.0	8.0	4	0.196
Lunasin 50 uM	12.5	16.4	3.9	4.7	9.4	6.7	3.4	4	0.968	90.8	110.1	94.9	116.0	102.9	12.0	6.0	4	0.691

PAI-1_TNF-a 10 ng/ml

PAI-1	ng/ml				mean	SD	SEM	n	LSD_p value	% of control				mean	SD	SEM	n	LSD_p value
test	1	2	3	4						1	2	3	4					
control	16.9	22.3	10.7	13.5	15.9	8.3	4.2	4		100.0	100.0	100.0	100.0	100.0	0.0	0.0	4	
Lunasin 5 uM	17.9	18.5	10.2	13.3	15.0	7.5	3.8	4	0.747	106.3	82.8	95.1	98.3	95.6	9.7	4.9	4	0.536
Lunasin 25 uM	15.6	10.5	12.1		12.7	6.7	3.9	3	0.297	92.3	97.5	89.4		93.1	4.1	2.4	3	0.368

Lunasin 50 uM	15.6	10.3	12.8		12.9	6.8	3.9	3	0.323	92.2	95.9	94.4		94.2	1.9	1.1	3	0.447

PAI-1_50%RAW-CM

PAI-1	ng/ml				mean	SD	SEM	n	LSD_p value	% of control				mean	SD	SEM	n	LSD_p value
test	1	2	3	4						1	2	3	4					
control	163.0	130.7	131.1	140.0	144.7	73.6	42.5	4		100.0	100.0	100.0	100.0	100.0	0.0	0.0	4	
Lunasin 5 uM	110.0	129.6	128.2		122.6	61.9	35.8	3	0.092	67.5	98.9	91.5		86.0	16.4	9.5	3	0.117
Lunasin 25 uM	116.1	104.6	127.8		116.2	58.8	34.0	3	0.030	71.2	79.8	91.2		80.8	10.0	5.8	3	0.039
Lunasin 50 uM	101.9	105.3	104.0	135.7	113.9	59.0	34.1	4	0.009	62.5	80.5	79.3	96.9	79.8	14.0	7.0	4	0.022

PAI-1_LPS 1000 ng/ml

PAI-1	ng/ml				mean	SD	SEM	n	LSD_p value	% of control				mean	SD	SEM	n	LSD_p value
test	1	2	3	4						1	2	3	4					
control	17.2	23.9	7.9	8.2	14.3	9.2	4.6	4		100.0	100.0	100.0	100.0	100.0	0.0	0.0	4	
Lunasin 5 uM	17.8	18.5	6.7	7.7	12.7	7.9	3.9	4	0.726	103.9	77.5	84.7	93.4	89.9	11.4	5.7	4	0.091
Lunasin 25 uM	15.7	18.7	6.7	7.5	12.1	7.5	3.7	4	0.643	91.4	78.2	84.7	91.6	86.5	6.4	3.2	4	0.029
Lunasin 50 uM	16.2	17.3	6.4	7.3	11.8	7.3	3.6	4	0.590	94.7	72.6	80.8	89.1	84.3	9.7	4.8	4	0.013

C. leptin

leptin_DMEM1%FBS

leptin	pg/ml				mean	SD	SEM	n	LSD_p value	% of control				mean	SD	SEM	n	LSD_p value
test	1	2	3	4						1	2	3	4					
control	85.6	113.6	56.6	94.6	87.6	23.73	11.87	4		100.0	100.0	100.0	100.0	100.0	0.0	0.0	4	
Lunasin 5 uM	93.6	103.6	44.6	86.6	82.1	25.96	12.98	4	0.766	109.3	91.2	78.8	91.5	92.7	12.6	6.3	4	0.538
Lunasin 25 uM	40.6	77.6	45.6	89.6	58.6	23.98	11.99	4	0.201	47.4	68.3	80.6	94.7	72.8	20.0	10.0	4	0.033
Lunasin 50 uM	63.6	83.6	49.6	109.6	76.6	26.05	13.03	4	0.553	74.3	73.6	87.6	115.9	87.8	19.8	9.9	4	0.311

leptin_TNF-a 10 ng/ml

leptin	pg/ml				mean	SD	SEM	n	LSD_p value	% of control				mean	SD	SEM	n	LSD_p value
test	1	2	3	4						1	2	3	4					

control	~~814.6~~	111.6	100.6	63.6	272.6	361.92	181.0	4		100.0	100.0	100.0	100.0	100.0	0.0	0.0	4	
Lunasin 5 uM	~~694.6~~	104.6	87.6	71.6	239.6	303.63	151.8	4	0.815	85.3	93.7	87.1	112.6	94.7	12.5	6.2	4	0.540
Lunasin 25 uM	~~679.6~~	84.6	67.6	64.6	224.1	303.79	151.9	4	0.263	83.4	75.8	67.2	101.6	82.0	14.6	7.3	4	0.051
Lunasin 50 uM	~~591.6~~	78.6	73.6	49.6	198.4	262.47	131.2	4	0.167	72.6	70.4	73.2	78.0	73.6	3.2	1.6	4	0.007

leptin_50%RAW-CM

leptin	pg/ml				mean	SD	SEM	n	LSD_p value	% of control				mean	SD	SEM	n	LSD_p value
test	1	2	3	4						1	2	3	4					
control	232.7	236.0	301.0	384.3	288.5	71.21	35.61	4		100.0	100.0	100.0	100.0	100.0	0.0	0.0	4	
Lunasin 5 uM	199.3	241.0	411.0		265.6	98.52	56.88	3	0.957	85.7	102.1	106.9		91.2	16.8	8.4	3	0.942
Lunasin 25 uM	299.3	227.7	362.7	396.0	321.4	74.25	37.13	4	0.689	128.7	96.5	120.5	103.0	112.2	15.0	7.5	4	0.592
Lunasin 50 uM	261.0	334.3	444.3	44.3	271.0	168.85	84.43	4	0.831	112.2	141.7	147.6	11.5	103.2	63.1	31.5	4	0.886

leptin_LPS 1000 ng/ml

leptin	pg/ml			mean	SD	SEM	n	LSD_p value	% of control			mean	SD	SEM	n	LSD_p value
test	1	2	3						1	2	3					
control	114.6	143.6	62.6	106.9	41.04	23.69	3		100.0	100.0	100.0	100.0	0.0	0.0	3	
Lunasin 5 uM	77.6	111.6	64.6	84.6	24.27	14.01	3	0.331	67.7	77.7	103.2	82.9	18.3	10.6	3	0.161
Lunasin 25 uM	64.6	75.6	47.6	62.6	14.11	8.14	3	0.072	56.4	52.6	76.0	61.7	12.6	7.3	3	0.008
Lunasin 50 uM	68.6	13.6	38.6	40.3	27.54	15.90	3	0.056	59.9	9.5	61.7	43.7	29.6	17.1	3	0.012

D. adiponectin

adiponectin_DMEM1%FBS

adiponectin	ng/ml					mean	SD	SEM	n	LSD_p value	% of control					mean	SD	SEM	n	LSD_p value
test	1	2	3	4	5						1	2	3	4	5					
control	537.4	481.2	400.3	424.1	659.6	500.5	103.6	46	5		100.0	100.0	100.0	100.0	100.0	100.0	0.0	0.0	5	
Lunasin 5 uM	347.0	366.0	305.5		664.1	420.6	164.2	82	4	0.402	64.6	76.1	76.3		100.7	79.4	15.2	7.6	4	0.050
Lunasin 25 uM	384.1	391.2	325.5	367.9	708.5	435.5	154.8	69	5	0.467	71.5	81.3	81.3	86.8	107.4	85.7	13.4	6.0	5	0.137
Lunasin 50 uM		398.4	299.3	476.0	633.5	451.8	141.1	71	4	0.607		82.8	74.8	112.2	96.0	91.5	16.4	8.2	4	0.393

adiponectin_TNF-a 10 ng/ml

adiponectin	ng/ml					mean	SD	SEM	n	LSD_p value	% of control					mean	SD	SEM	n	LSD_p value
test	1	2	3	4	5						1	2	3	4	5					
control	366.5	323.6	293.1	171.7	358.5	302.7	78.8	35	5		100.0	100.0	100.0	100.0	100.0	100.0	0.0	0.0	5	
Lunasin 5 uM	337.4	217.9	190.8	235.5	328.5	262.0	66.8	30	5	0.388	92.1	67.3	65.1	137.2	91.6	90.7	29.0	13.0	5	0.636
Lunasin 25 uM	275.5	299.3	159.3	171.7	310.2	243.2	72.2	32	5	0.212	75.2	92.5	54.4	100.0	86.5	81.7	17.8	8.0	5	0.357
Lunasin 50 uM	258.4	278.4	128.4	264.6	319.1	249.8	71.8	32	5	0.265	70.5	86.0	43.8	154.1	89.0	88.7	40.7	18.2	5	0.566

adiponectin_50%RAW-CM

adiponectin	ng/ml					mean	SD	SEM	n	LSD_p value	% of control					mean	SD	SEM	n	LSD_p value
test	1	2	3	4	5						1	2	3	4	5					
control	281.2	327.9	236.0	163.1	279.6	257.6	62.0	28	5		100.0	100.0	100.0	100.0	100.0	100.0	0.0	0.0	5	
Lunasin 5 uM	302.2	230.3	187.4	207.9	270.2	239.6	46.5	21	5	0.628	107.5	70.2	79.4	127.4	96.6	96.2	22.7	10.1	5	0.820
Lunasin 25 uM	272.2	200.3	160.3	210.8	308.5	230.4	59.3	27	5	0.466	96.8	61.1	67.9	129.2	110.3	93.1	28.6	12.8	5	0.677
Lunasin 50 uM	254.6	281.7	143.6	229.3	290.7	240.0	59.0	26	5	0.635	90.5	85.9	60.9	140.6	104.0	96.4	29.2	13.1	5	0.827

adiponectin_LPS 1000 ng/ml

adiponectin	ng/ml					mean	SD	SEM	n	LSD_p value	% of control					mean	SD	SEM	n	LSD_p value
test	1	2	3	4	5						1	2	3	4	5					
control	381.2	272.7	365.5	447.0	532.9	399.9	97.0	43	5		100.0	100.0	100.0	100.0	100.0	100.0	0.0	0.0	5	
Lunasin 5 uM	378.9	275.0	366.5	429.8	597.9	409.6	119.2	53	5	0.897	99.4	100.9	100.3	96.2	112.2	101.8	6.1	2.7	5	0.888
Lunasin 25 uM		309.8	301.2	434.1	620.2	416.3	148.8	74	4	0.837		113.6	82.4	97.1	116.4	102.4	15.8	7.9	4	0.861
Lunasin 50 uM	404.1	438.9	285.0	543.1	579.6	450.2	117.2	52	5	0.508	106.0	161.0	78.0	121.5	108.8	115.0	30.2	13.5	5	0.248
